# Equilibrium Structures of Propane and 2,2-Difluoropropane and Comparison with Other Two-Top Molecules

**DOI:** 10.3390/molecules29204877

**Published:** 2024-10-14

**Authors:** Jean Demaison, Natalja Vogt, Agnès Perrin

**Affiliations:** 1 Physique des Lasers, Atomes et Molécules, Université de Lille, Bât. P5, CEDEX, 59655 Villeneuve d’Ascq, France; 2 Faculty of Sciences, University of Ulm, 89069 Ulm, Germany; natalja.vogt@alumni.uni-ulm.de; 3 Laboratoire de Météorologie Dynamique/IPSL CNRS, Ecole Polytechnique, RD36, CEDEX, 91128 Palaiseau, France; agnes.perrin@lmd.ipsl.fr

**Keywords:** equilibrium structure, semiexperimental, ab initio, propane

## Abstract

The Born–Oppenheimer ab initio equilibrium structures of propane (CH_3_)_2_CH_2_ and 2,2-difluoropropane (CH_3_)_2_CF_2_ were computed at the CCSD(T) level of theory using a basis set of quadruple zeta quality. The semiexperimental structure of propane was also determined from the ground state rotational constants corrected for rovibrational corrections calculated at the MP2 level of theory. Structural comparisons are made with other molecules and are discussed in terms of the quantum theory of atoms in molecules.

## 1. Introduction

**Figure 1:**
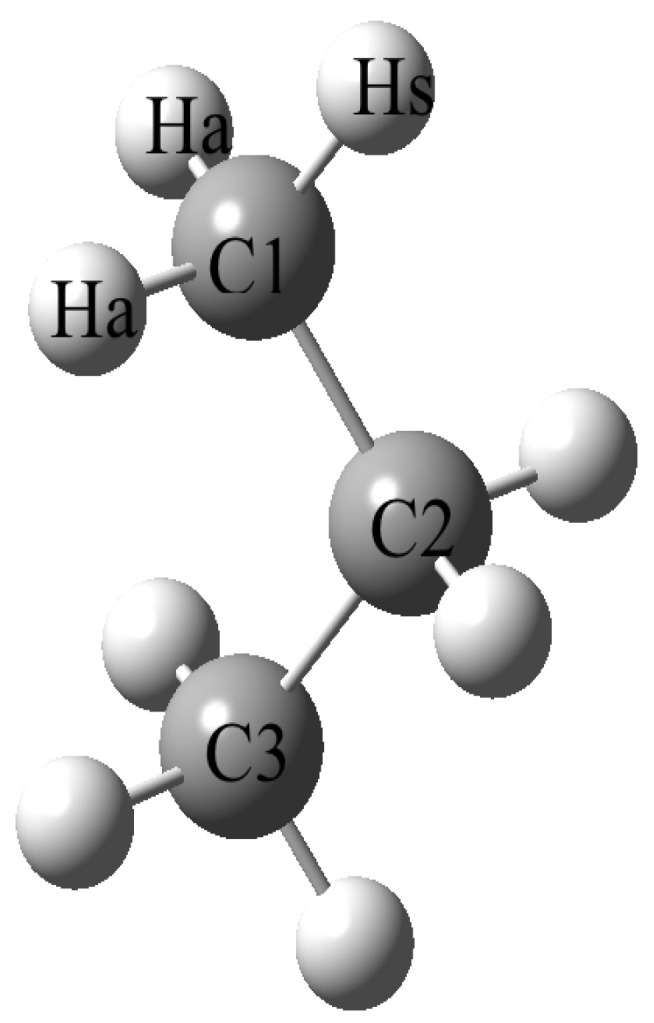
Structure of propane.

Propane, C_3_H_8_, is one of the simplest acyclic saturated hydrocarbons; see Figure 1. It is a pollutant of the earth’s atmosphere as it is produced by biomass burning. Propane was also found in the atmosphere of the satellite Titan [1]. It is mainly used as fuel, in particular as liquefied petroleum gas (LPG).

The propane molecule is an asymmetric top of C_2v_ symmetry. Despite its small dipole moment, the microwave spectrum of propane in the ground vibrational state was measured early by Lide [2], who determined the dipole moment and a substitution structure (*r*_s_) of this molecule. Later, the accuracy of the ground state rotational constants was improved [3,4]. The internal rotation of the methyl groups was studied in the vibrational ground state [4,5] as well as in the microwave spectra of the torsional excited states [6,7,8]. The internal rotation was also investigated by Raman spectroscopy [9,10,11] and by inelastic neutron scattering [12]. There are also several high-resolution infrared studies [13,14,15,16,17].

The goal of this paper is first to determine an accurate equilibrium structure of propane using the semiexperimental method and, as a check, high-level ab initio calculations. Then, this structure is compared to those of two-top molecules whose equilibrium structures are known.

The equilibrium structure corresponds to the minimum of the potential hypersurface. It does not depend on the temperature or the isotopic substitutions [18,19]. It is obtained by high-level ab initio optimizations. It can also be determined experimentally from a fit of the equilibrium rotational constants (or the corresponding moments of inertia). The equilibrium rotational constants, *B*_e_, are obtained by correcting the ground state rotational constants, *B*_0_, from the rovibrational contribution. As this correction is difficult to obtain experimentally, it is easier to calculate from an ab initio cubic force field, giving the semiexperimental equilibrium structure. This semiexperimental structure is known to be often the most accurate one [18]. Indeed, Bak et al. [20] and Pawłowski et al. [21] made a systematic analysis of the semiexperimental structure of nearly 20 small molecules and concluded that the equilibrium bond distances determined by the semiexperimental method surpass the accuracy obtainable either by purely experimental techniques (except for the smallest systems, such as diatomic molecules) or by ab initio methods.

Many empirical methods have also been proposed to estimate the equilibrium structure [19]. The simplest one, yielding the effective structure, *r*_0_, assumes that the equilibrium rotational constants are identical to the ground state rotational constants. Although the rovibrational correction is only a few percent of the rotational constants, the *r*_0_ structure can be a poor approximation of the equilibrium structure. A slightly better method, yielding the substitution structure, *r*_s_, assumes that the rovibrational correction is isotopically independent. In this case, the difference between the moment inertia of an isotopologue and the parent species is used. A better approximation is the $r_{m}^{\rho }$ method where the structural parameters are fitted to the moments of inertia $I_{m,g}^{\rho }=\left(2\rho_{g}-1\right)I_{g}^{0}(i)$, with $\rho_{g}=I_{g}^{s}(1)/I_{g}^{0}(1)$, where 1 is for the parent isotopologue and *g* = *a*, *b*, *c*. Finally, the *r*_z_ (=$r_{\alpha }^{0}$) distance is the distance between the average nuclear positions in the vibrational ground state at 0 K. It has a clear physical meaning permitting comparisons between molecules. It is often a good approximation of the equilibrium bond angles.

The first structure determinations of propane were carried out by gas-phase electron diffraction [22,23] about ninety years ago. Later, the traditional set of structural parameters (thermal-average internuclear distances *r*_g_ and angles ∠*_α_*) as well as a zero-point average structure (*r*_z_) were determined by Iijima [24] from electron diffraction data as well as by their combination with zero-point moments of inertia derived from microwave data [2]. Using the experimental data of Lide [2], Tam et al. [25] calculated empirical *r*_0_, *r*_s_, and $r_{m}^{\rho }$ structures, the last one being often a good approximation of the equilibrium structure. Finally, an ab initio equilibrium structure was calculated at the CCSD(T)/cc-pVQZ level of theory by Villa et al. [26].

As a complement, for the sake of comparison, the equilibrium structure of 2,2-difluoropropane, (CH_3_)_2_CF_2_, is also calculated by an ab initio method. For this molecule, empirical *r*_0_ and *r*_s_ structures were determined by Takeo et al. [27], and the *r*_a_/∠*_α_* structure was also obtained by Mack et al. [28] by a joint analysis of electron diffraction intensities and rotational constants.

## 2. Computational Methods

Different quantum-mechanical methods were used in the present study. Ab initio computations were carried out at two levels: second-order Møller–Plesset perturbation theory (MP2) [29] and coupled cluster (CC) theory, with single and double excitation [30], augmented by a perturbational estimate of the effects of connected triple excitations [CCSD(T)] [31]. The Kohn–Sham density functional theory (DFT) [32] was also used with one hybrid Becke functional [33] and with Lee–Yang–Parr non-local correlation (B3LYP) [34]. Several basis sets were used, including Pople’s 6-311+G(2d,2p) [35], the correlation-consistent polarized *n*-tuple zeta cc-pV*n*Z [36], and the correlation-consistent polarized weighted core-valence *n*-zeta cc-pwCV*n*Z [37]. The CCSD(T) calculations were performed with the MOLPRO [38,39] program, while other calculations utilized the Gaussian09 program package [40]. Finally, the Atom in Molecules (AIM) theory [41,42] with its implementation in Gaussian by Cioslowski et al. [43,44,45,46,47,48] was used.

## 3. Equilibrium Structures

### 3.1. Ab Initio Structure

First, in order to obtain reliable predicate values, the ab initio structure was optimized at the CCSD(T)_AE/cc-pwCVQZ level of theory, all electrons being correlated (AE). This level of theory is expected to give a result close to the Born–Oppenheimer equilibrium structure ($r_{e}^{\mathrm{BO}}$) [49,50]. This structure is given in Table 1. The computed ab initio structures are given in Table S1 of the Supplementary Materials. For the structure optimization with Gaussian, the tight option was used, and in Molpro, the criterium GRAD = 5 was employed.

### 3.2. Semiexperimental Equilibrium Structure of Propane

To correct the effective experimental rotational constants for each isotologue and to obtain their equilibrium counterparts, anharmonic (up to semidiagonal quartic terms) force field computations were performed for the structure optimized at the level of second-order Møller–Plesset perturbation theory [29], MP2, using the standard correlation consistent valence triple-ζ basis set [36].

The semiexperimental equilibrium rotational constants, *B*_e_, can be calculated from the experimental ground state rotational constants, *B*_0_, using the following equation:

*B*_e_ = *B*_0_ + ∆*B*_vib_ + ∆*B*_el_ (1)

where ∆*B*_vib_ is the rovibrational correction calculated from the cubic force field, and ∆*B*_el_ is the electronic correction, which may be obtained from the rotational *g* tensor. In theory, there is also a small centrifugal distortion correction, but, in the present case, it was found quite small (1 kHz for *A*, 25 kHz for *B*, and −23 kHz for *C*) and was neglected in the final fits. The experimental g-constants were determined by Zeeman spectroscopy and are taken from Ref. [51]. In this particular case, the electronic correction happens to be small (−0.97 MHz for *A*; −28 kHz for *B*; and negligible for *C*).

For the ground state rotational constants of the parent species, the values of Drouin et al. [4] were used. It is known that accurate ground state rotational constants are valuable for structure determination [52]. For this reason, the transitions of the isotopologues measured by Lide [2] were refitted using the mixed estimation method, in which theoretical centrifugal distortion constants derived from the ab initio quadratic MP2/cc-pVTZ force field are used as supplementary data in a weighted least-squares fit to the transitions [53,54]. The starting uncertainties of these predicate centrifugal distortion constants were 10% of their values. The rovibrational corrections and the semiexperimental rotational constants are given in Table 2. The pseudo-inertial defect ∆_se_ = *I_c_* − *I_b_* − *I_a_* is also given in this Table. It is almost constant for the isotopologues, which keep the symmetry *C*_2v_, indicating that the corrections to the rotational constants are consistent. The equilibrium structural parameters were determined by a weighted least-squares fit based on the semiexperimental equilibrium moments of inertia. The weights of the semiexperimental moments of inertia were determined iteratively. At each step, an analysis of the residuals permitted checking the appropriateness of the weights. It is possible to automate, at least partly, this procedure by using the Iteratively Reweighted Least-Squares (IRLS) method, whereby data with large residuals are weighted down [55]. The biweight weighting scheme was used, where the weight decreases as the residual increases and where data with large residuals are eliminated.

It is also possible to estimate the CH bond lengths using the relationship between the isolated stretching frequency and the CH bond length [19]. The results are also given in Table 1. The equilibrium values of the Cartesian coordinates are given in Table S2.

The semiexperimental $r_{e}^{se}$, ab initio $r_{e}^{\mathrm{BO}}$, and $r_{m}^{\rho }$ structures compared in Table 1 are consistent, although the $r_{m}^{\rho }$ structure is obviously less accurate. Finally, the CH bond lengths derived from the isolated stretching frequencies are in excellent agreement with the results of the other methods.

## 4. Discussion

### 4.1. Comparison with the Internal Rotation Parameters

The structural parameters derived from the internal rotation analysis are the moment of inertia of the top, *I_α_*, and the angles between the axis of rotation, *i*, of the top and the principal axes of the molecule, ∠(*i*,*g*), with *g* = *a*, *b*, *c*. The determined values are given in Table 3, where they are compared with the values of the equilibrium structure and with the values of some other two-top molecules.

*I_α_* is highly correlated with the potential barrier, and, thus, it is difficult to be determined. Furthermore, the result depends on the method of analysis used. This difficulty was analyzed by Bauder and Günthard in the particular case of the ground state of acetaldehyde CH_3_CHO [62]. They found that *I_α_* varies between 3.1975 uÅ^2^ and 3.2453 uÅ^2^ depending on the method of analysis used. IIjima and Tsuchiya showed that *I_α_* is particularly sensitive to vibrational averaging, which increases its effective value [63]. For propane, *I_α_* is larger by 0.067 uÅ^2^ than the value derived from the structure. Indeed, a comparative study of the methyl internal rotation in 17 different molecules concluded that this difference is rather systematic, with a median value of 0.077 (11) uÅ^2^ (the mean being almost identical: 0.080 uÅ^2^) [64].

On the other hand, the angles ∠(*i*,*g*) can be determined with high accuracy, but they are also vibrationally averaged parameters, and it is not obvious that the internal rotation axis *i* coincides with the bond axis [19]. As shown in Table 3, the values of the ∠(*i*,*g*) angles are not compatible with the values of the equilibrium structure. This discrepancy is called methyl tilt [65]. The vibrational dependence of the angle seems to be large. The data are not numerous but, for instance, for methyl glycolate, CH_3_OC(O)CH_2_OH, ∠(*i*,*a*) = 26.3 (6)° for the ground state, 21.3 (5)° for the first torsional state, and 23.5 (8)° for the CO torsion [66]. Likewise, for dimethyl selenide, (CH_3_)_2_Se, ∠(*i*,*b*) = 50.31 (6)° for the vibrational ground state, but 42.35 (5)° for the first excited state of the CSeC bend [67].

The conclusion is that these two structural parameters are fitting parameters, which can be quite different from the values of the structure.

### 4.2. Comparison of the CC Bond Lengths

The CC bond length in propane at 1.522 (1) Å is almost identical to the value found for ethane; CH_3_CH_3_: 1.522 (2) Å [68]. On the other hand, the CC bond length in 2,2-difluoropropane at 1.514 Å is significantly shorter. It is known that the substitution of a hydrogen by fluorine on one carbon shortens the CC bond [28]. A few typical examples are given in Table S3 for the single, double, and triple CC bonds and also for the double CO bonds. It may be explained by the high electronegativity of fluorine, which is, therefore, an electron-withdrawing substituent. Hence, the carbon atom in 2,2-difluoropropane is much more positive than in propane: an AIM calculation at the B3LYP/6-311+(2f,2d) level gives for the charge *q*(C2), 0.0949 au for propane, and 1.071 au for 2,2-difluoropropane (a complete list of charges is given in Table S4). The consequence is that the atomic radius is much smaller in 2,2-difluoropropane, explaining the shorter bond length [42]. Another typical result is the increase of the CCC bond angle from 112° in propane to 115.9° in 2,2-difluoropropane. As the CC bond length is shorter in 2,2-difluoropropane, the larger bond angle permits to keep about the same non-bonded distance between the two out-of-plane hydrogens on different carbons: 2.593 Å in propane and 2.635 Å in 2,2-difluoropropane.

### 4.3. Comparison of the CF Bond Lengths

The CF bond length in 2,2-difluoropropane at 1.369 Å is slightly shorter than in methyl fluoride, CH_3_F, for which the value is 1.383(1) Å [69]. It may be explained by the smaller atomic radius of the carbon atom in 2,2-difluoropropane, which has a much larger positive charge than in methyl fluoride (1.071 au for the former and 0.640 au for the latter), whereas the charges on the fluorine atom are similar (−0.660 au for 2,2-difluoropropane and −0.645 au for methyl fluoride). For 2,2-difluoropropane, the product *q*(F) × *q*(C) is large in absolute value, indicating a significant ionic character [70].

### 4.4. Geometry of the Methyl Group

Although the CH_s_ and CH_a_ bond lengths are close in propane, 1.089 Å and 1.091 Å, respectively, the methyl group is asymmetric, and the ∠(H_a_C2C1) bond angle is slightly larger than the ∠(H_s_C2C1) bond angle. This is usual for an asymmetric top, and the consequence is a methyl tilt. The CH bond lengths and ∠(HXH) bond angles (with X = C, O, S being the central atom) are compared for a few molecules in Table 4.

According to the ligand close-packing model [42], the distance between two non-bonded atoms attached to the same atom is nearly constant and is the sum of the ligand radii of the two atoms. Actually, the ligand radius depends on the charge of the atom, and it decreases as the charge becomes less negative (or more positive). For the H_a_^…^H_a_ distance, it is rather well verified. For a selection of nine molecules, the Spearman rank correlation coefficient is 0.88; see Table 4. However, other factors apart from the difference of charge are not negligible.

Likewise, as a consequence, there is a nice correlation between the H_a_^…^H_a_ distance and the ∠(HaCHa) angle, with a Spearman rank correlation coefficient of 0.92; see Table 4. This correlation is well established and has been first discussed by McKean for the methyl group [72] and by Demaison et al. [73,74] for the methylene group.

A similar correlation is found between the H_s_^…^H_a_ distance and the H_s_CH_a_ bond angle, with a correlation coefficient of 0.94; see Table 4. It has to be noted that the oxygen atom in (CH_3_)_2_O has an important negative charge of −1.084 au and that both hydrogen atoms are rather close to the oxygen atom: 2.078 Å for H_a_ and 2.020 Å for H_s_, with H_a_ having a negative charge of −0.026 ua and H_s_ having a small positive charge of 0.0001 ua. This corresponds to the sum of the ligand radii: *r*(H) = 0.82 Å and *r*(O) = 1.25 Å [42]; see Table S5 of the Supplementary Materials. In this particular case, apart from the interaction between the hydrogen atoms, there is also an interaction between the hydrogen atoms and the oxygen atom. In this simple case of (CH_3_)_2_O, the interactions between the hydrogen atoms and the oxygen atom may explain the asymmetry of the methyl groups.

## 5. Conclusions

The semiexperimental structure of propane has been determined, and, as the check, the structure was also optimized at the CCSD(T)_ae/cc-pwCVQZ level of theory. In addition, the structure of 2,2-difluoropropane was also optimized at the same level of theory. The structure of propane is used to calculate the moment of inertia of the methyl tops and the angle between the axis of internal rotation and the principal axes of the molecule. These two parameters are also obtained from the analysis of internal rotation splittings of the rotational spectrum. The agreement is not good and is explained by the fact that the internal rotation parameters are vibrationally averaged and, thus, different from the equilibrium values.

The variation in the CC, CF, and CH bond lengths in different molecules is also discussed using the AIM (Atom In Molecule) theory and is explained by the variation of the atomic radius with the electronegativity of the substituents.

## Figures and Tables

**Table 1:** Equilibrium structures of propane and 2,2-difluoropropane (distances in Å; angles in degrees).

(CH_3_)_2_CH_2_						(CH_3_)_2_CF_2_
	$r_{m}^{\rho }$ [21]	$\mathrm{Semiexperimental}\;r_{e}^{se}$		From ν^is d^		
		Weighted Fit ^b^	IRLS ^c^		$r_{e}^{\mathrm{BO}}$ ^e^	$r_{e}^{\mathrm{BO}}$ ^e^
C1C2	1.5209 (9)	1.52226 (6)	1.52207 (10)		1.5226	1.5143
C1H_s_	1.0877 (35)	1.08940 (22)	1.08916 (42)	1.089	1.0894	1.0869
C1H_a_	1.0907 (19)	1.09087 (10)	1.09113 (23)	1.090	1.0908	1.0873
C2Y ^a^	1.0929 (20)	1.09184 (15)	1.09227 (25)	1.091	1.0917	1.3691
C1C2C3	112.35 (11)	112.070 (6)	112.089 (13)		112.10	115.89
C2C1H_s_	111.60 (31)	111.656 (14)	111.687 (36)		111.70	109.10
C2C1H_a_	110.62 (10)	110.745 (16)	110.752 (12)		110.79	109.85
H_s_C1H_a_		107.950 (17)	107.952 (26)		107.91	109.36
H_a_C1H_a_	107.04 (28)	107.642 (31)	107.589 (33)		107.58	109.32
YC2Y ^a^	106.13 (32)	106.448 (44)	106.419 (39)		106.38	105.75
YC2C1H_a_ ^a^		59.671 (16)	59.643 (21)		59.66	60.140

^a^ Y = H for propane or Y = F for 2,2-difloropropane. See also Figure 1 for the notations. ^b^ Uncertainties used for the weighting (in MHz): 0.3 for *A* and 0.1 for *B* and *C*. ^c^ Iteratively reweighted least-squares fit; see text. ^d^ From isolated stretching frequencies; see text. ^e^ CCSD(T)_AE/cc-pwCVQZ optimization.

**Table 2:** Experimental ground state rotational constants (*A*_0_, *B*_0_, *C*_0_), rovibrational corrections (*A*_e_ − *A*_0_, *B*_e_ − *B*_0_, *C*_e_ − *C*_0_), semiexperimental equilibrium rotational constants (*A*_se_, *B*_se_, *C*_se_), and pseudo-inertial defect ∆_se_ = *I_c_* − *I_b_* − *I_a_*, residuals of the fit (*A*_se_ − *A*_calc_, *A*_se_ − *A*_calc_, *C*_se_ − *C*_calc_) for propane. Values in MHz, except for ∆ in uÅ^2 a^.

	Parent	^13^C1	^13^C2	CH_2_Ds	CH_2_Da	CHD(CH_3_)_2_
*A*_0_	29,207.47	29,092.14	28,660.68	29,017.92	26,829.12	25,830.05
*B*_0_	8445.97	8228.75	8446.84	7838.19	8122.83	8358.63
*C*_0_	7459.00	7281.77	7423.14	6971.95	7185.07	7282.97
*A*_e_ − *A*_0_	293.22	290.82	281.54	302.07	260.18	244.08
*B*_e_ − *B*_0_	104.78	101.36	103.96	94.06	98.18	103.03
*C*_e_ − *C*_0_	95.20	92.32	93.97	85.03	89.70	91.49
*A*_se_	29,499.72	29,381.99	28,941.28	26,073.45	29,319.05	27,088.51
*B*_se_	8550.72	8330.08	8550.78	8461.63	7932.22	8220.98
*C*_se_	7554.20	7374.09	7517.11	7374.46	7056.98	7274.77
∆	−9.335	−9.335	−9.335	−9.335	−10.661	−10.578
*A*_se_ − *A*_calc_	−0.34	−0.07	0.16	0.15	0.01	0.11
*B*_se_ − *B*_calc_	−0.06	0.05	−0.01	−0.05	−0.04	0.10
*C*_se_ − *C*_calc_	0.00	0.11	0.08	−0.15	0.07	−0.09

^a^ For the notation of the atoms, see Figure 1.

**Table 3:** Structural parameters of internal rotation analysis, ir, compared to the values of the semiexperimental equilibrium structure, se (moments of inertia *I_α_* in uÅ^2^, angles ∠ in degrees).

	Internal Rotation Analysis			Equilibrium Structure				
	$I_{\alpha }^{ir}$	∠(*i*,*i*) ^a^	Ref.	$I_{\alpha }^{se}$	∠(CXC)	Ref.	Tilt ^b^	∆*I_α_* ^c^
(CH_3_)_2_CH_2_	3.198 (21)	109.70 (13)	[5]	3.131	112.09 (1)	This work	2.4	0.067
(CH_3_)_2_O	3.263 (7)	118.00 (18)	[56]	3.186	111.10 (3)	[57]	−6.9	0.077
(CH_3_)_2_S	3.225 (7)	103.40 (1)	[58]	3.184	98.58 (1)	[59]	−4.8	0.041
(CH_3_)_2_CO	3.215 (6)	120.50 (12)	[60]	3.162	116.5 (1)	[61]	−4.0	0.053

^a^ Angle between the two internal rotation axes. ^b^ Methyl tilt: ∠(CXC)−∠(*i*,*i*). ^c^ ∆I_α_ = $I_{\alpha }^{ir}-I_{\alpha }^{se}$.

**Table 4:** Charge *q* (au) on the out-of-plane (a) hydrogen atoms and distance *d* (Å) between the hydrogen atoms and angle ∠(HXH) between the hydrogen atoms (degree), with X being the central atom: X = C, O, S ^a^.

	*q*(H_a_) ^b^	*d*(H_a_^…^H_a_)	∠(H_a_XH_a_)	*d*(H_s_…H_a_)	∠(H_s_XH_a_)	Ref.
CH_4_	−0.01388	1.7737	109.47	1.7737	109.47	[71]
CH_3_F	0.01554	1.7837	110.26	1.7837	110.26	[69]
CH_3_CH_3_	−0.02773	1.7579	107.67	1.7579	107.67	[68]
(CH_3_)_2_CH_2_ ^c^	−0.02986	1.7602	107.64	1.7628	107.95	This work
(CH_3_)_2_CH_2_ ^d^	−0.03627	1.7480	106.38			This work
(CH_3_)_2_O	−0.01923	1.7780	108.55	1.7780	109.18	[57]
(CH_3_)_2_S	0.00416	1.7823	109.73	1.7748	109.13	[59]
(CH_3_)_2_CO	−0.03902	1.7554	107.04	1.7821	109.97	[61]
(CH_3_)_2_CF_2_	0.00471	1.7738	109.32	1.7740	109.36	This work

^a^ Semiexperimental structures, except for CH_3_CH_3_, whose structure was optimized at the CCSD(T)/cc-pV∞Z level of theory. ^b^ B3LYP/6-311+G(2d,2p) level of theory. ^c^ Methyl group. ^d^ Methylene group.

## Data Availability

All new data are either in the main text or in the Supplementary Materials.

## Supplementary Materials

The following supporting information can be downloaded at: https://www.mdpi.com/article/10.3390/molecules29204877/s1, Table S1: Computed ab initio structures of propane (in Å). Table S2: Cartesian coordinates for the atoms of propane in the principal axes system (in Å). Table S3: Effect of fluorination on the carbon-carbon bond length (Å) in some molecules, experimental or semiexperimental equilibrium structures unless otherwise stated. Table S4: Charges (au) on the atoms of propane and 2,2-difluoropropane computed at the B3LYP/6-311+G(2d,2p). Table S5: Experimental or semiexperimental equilibrium structure of the HCO moiety in some molecules (distances in Å. angles in degree). Refs. [57,74,75,76,77,78,79] are cited in the Supplementary Materials file.
