# Supplementary material for: Equilibrium Structures of Propane and 2,2-Difluoropropane and Comparison with Other Two-Top Molecules
Source: Molecules. 2024 Oct 14;29(20):4877. doi: 10.3390/molecules29204877 (PMC11510024; doi:10.3390/molecules29204877)
Supplement: Supplementary file 1 [file molecules-29-04877-s001.zip › molecules-3211223-supplementary.pdf]

molecules

Equilibrium Structures of Propane and 2,2-Difluoropropane and Comparison with Other Two-Top Molecules

Jean Demaison [1,*], Natalja Vogt [2] and Agnès Perrin [3]

[1] Physique des Lasers, Atomes et Molécules, Université de Lille, Bât. P5, 59655 Villeneuve d'Ascq CEDEX, France
[2] Faculty of Sciences, University of Ulm, 89069 Ulm, Germany; natalja.vogt@alumni.uni-ulm.de
[3] Laboratoire de Météorologie Dynamique/IPSL CNRS, Ecole Polytechnique, RD36, 91128 Palaiseau Cedex, France; agnes.perrin@lmd.ipsl.fr
* Correspondence: jean.demaison@gmail.com

Table S1. Computed *ab initio* structures of propane (in Å).

	**CCSD(T)_AE**	**MP2**	**B3LYP**
	cc-pwCVQZ	cc-pVTZ	6-311(3DF.2pd)
C1C2	1.5226	1.5230	1.5281
C1Hs	1.0894	1.0887	1.0908
C1Ha	1.0908	1.0900	1.0931
C2H	1.0917	1.0909	1.0931
C1C2C3	112.10	111.93	112.97
C2C1Hs	111.70	111.79	111.56
C2C1Ha	110.79	110.73	111.10
HsC1Ha	107.91	107.91	107.70
HaC1Ha	107.58	107.60	107.50
HC2H	106.38	106.42	106.07
XC2C1Ha	59.66	59.63	59.81

Table S2. Cartesian coordinates for the atoms of propane in the principal axes system (in Å).

	**Semiexperimental structure**			CCSD(T)_ae/cc-pwCVQZ		
	a	***b***	***c***	*a*	*b*	*c*
C1	1.26252(5)	-0.26972(5)	0	1.2814	-0.27266	0
H1s	2.16163(16)	0.34542(31)	0	2.1644	0.35976	0
H1a-	1.29519(25)	-0.91285(21)	-0.88052(18)	1.3185	-0.90859	-0.87962
H1a+	1.29519(25)	-0.91285(21)	0.88052(18)	1.3185	-0.90859	0.87962
C2	0	0.58054(6)	0	0	0.58352	0
H(C2)	0	1.23442(25)	-0.87454(35)	0	1.22999	-0.87583
H(C2)	0	1.23442(25)	0.87454(35)	0	1.22999	0.87583
C3	-1.26252(5)	-0.26972(5)	0	-1.2814	-0.27266	0
H3s	-2.16163(16)	0.34542(31)	0	-2.1644	0.35976	0
H3a+	-1.29519(25)	-0.91285(21)	0.88052(18)	-1.3185	-0.90859	0.87962
H3a-	-1.29519(25)	-0.91285(21)	-0.88052(18)	-1.3185	-0.90859	-0.87962

Table S3. Effect of fluorination on the carbon-carbon bond length (Å) in some molecules, experimental or semiexperimental equilibrium structures unless otherwise stated.

CH_3CH_3	CH_3CH_2F	CH_3CHF_2
1.536 [a]	1.512(2) r_s [b]	1.501(10) r_0 [c]
$CH_3CH_2CH_3$	CH_3CHFCH_3	$CH_3CF_2CH_3$
1.522 [d]	1.515(1) r_s [e]	1.514 [d]
$CH_2{=}CH_2$	$CH_2{=}CHF$	$CH_2{=}CF_2$
1.331 [f]	1.321 [g]	1.318 [h]
HCCH	HCCF	FCCF
1.203 [i]	1.196 [j]	1.197(3) [k]
c-C_3H_6		*c*-$C_3H_4F_2$
1.503 [l]		1.464 r_s [m]
c-C_6H_6	*c*-C_6H_5F	*o*-$C_6H_4F_2$
1.205 [n]	1.177 [n]	1.170 [n]
OCH_2	OCHF	OCF_2
1.205 [o]	1.177 [p]	1.170 [q]

[a] CCSD(T)/cc-pV∞Z. C. Puzzarini, P.R. Taylor. An ab initio study of the structure, torsional potential energy function, and electric properties of disilane, ethane, and their deuterated isotopomers. J. Chem. Phys. 122 (2005) 054315.

[b] M. Hayashi, M. Fujitake, T. Inagusa, S. Miyazaki. Microwave spectrum of ethyl fluoride and structure of CH_3MH_2X type molecules. J. Mol. Struct. 216 (1990) 9-26.

[c] R.M. Villamañan, W.D. Chen, G. Wlodarczak, J. Demaison, A.G. Lesarri, J.C. López, J.L. Alonso. Rotational Spectrum of 1, 1-Difluoroethane: Internal Rotation Analysis and Structure. J. Mol. Spectrosc. 171 (1995) 223-247.

[d] This work.

[e] H. Kakubari, T. Iijima, M. Kimura. Molecular structure of 2-fluoropropane as determined by gas electron diffraction. Bull. Chem. Soc. Japan 48 (1975) 1984-1986.

[f] J.M.L. Martin, T.J. Lee, P.R. Taylor, J.-P. François. The anharmonic force field of ethylene, C2H4, by means of accurate ab initio calculations. J. Chem. Phys. 103 (1995) 2589-2602.

[g] J. Demaison. Ab initio anharmonic force field and equilibrium structure of vinyl fluoride and vinyl iodide. J. Mol. Spectrosc. 239 (2006) 201-207.

[h] N. Vogt, J. Demaison, J. Vogt, H.D. Rudolph. Why it is sometimes difficult to determine the accurate position of a hydrogen atom by the semiexperimental method: structure of molecules containing the OH or the CH_3 group. J. Comput. Chem. 35 (2014) 2333-2342.

[i] F. Tamassia, E. Cané, L. Fusina, G. Di Lonardo. The experimental equilibrium structure of acetylene. Phys Chem Chem Phys 18 (2016) 1937-1944.

[j] P. Botschwina, S. Seeger. A "Bernstein-McKean-Duncan" plot and the equilibrium geometry of monofluoroacetylene. J. Mol. Struct. 320 (1994) 243-247.

[k] H. Bürger, M. Senzlober, S. Sommer. Ground state and equilibrium structure of FCCF from high-resolution IR spectra of $F^{12}C^{13}CF$. J. Mol. Spectrosc. 164 (1994) 570-573.

[l] CCSD(T)/cc-pVQZ. J. Gauss, D. Cremer, J.F. Stanton. The re structure of cyclopropane. J. Phys. Chem. A 104 (2000) 1319-1324.

[m] A.T. Perretta, V.W. Laurie. Microwave spectrum, structure, and dipole moment of 1,1-difluorocyclopropane. J. Chem. Phys. 62 (1975) 2469-2473.

[n] J. Demaison, H.D. Rudolph, A.G. Császár. Deformation of the benzene ring upon fluorination: equilibrium structures of all fluorobenzenes. Mol. Phys. 111 (2013) 1539-1562.

[o] W.J. Morgan, D.A. Matthews, M. Ringholm, J. Agarwal, J.Z. Gong, K. Ruud, W.D. Allen, J.F. Stanton, H.F. Schaefer III. J. Chem. Theory Comput. 14 (2018) 1333-1350.

[p] L. Margulès, J. Demaison, J.E. Boggs. Equilibrium C-F bond length and the structure of formyl fluoride, difluorocarbene, monofluoromethylene and difluoromethane. J. Phys. Chem. A 103 (1999) 7632–7638.

[q] J. Demaison, A.G. Császár. Equilibrium CO bond lengths. J. Mol. Struct. 1023 (2012) 7-14.

Table S4. Charges (au) on the atoms of propane and 2,2-difluoropropane computed at the B3LYP/6-311+G(2d,2p).

**Propane**		2.2-difluoropropane	
**C1**	**0.0706**	C1	0.0751
C2. C3	0.0949	C2. C3	1.0711
H (CH_2)	-0.0363	F	-0.6453
Hs	-0.0272	Hs	0.0199
Ha	-0.0299	Ha	0.0047

Table S5. Experimental or semiexperimental equilibrium structure of the HCO moiety in some molecules (distances in Å. angles in degree).

Molecule	*r*(CH)	*r*(CO)	∠(HCO)	*d*(H···O)	*q*(O)	Ref.
CH_3OH s	1.086	1.417	106.79	2.019	-1.107	1
CH_3OH a	1.091	1.417	111.95	2.087	-1.107	1
OCH_2	1.101	1.205	121.65	2.013	-1.099	2
OCHCl	1.092	1.182	126.30	2.029	-1.104	3
OCHF	1.090	1.177	127.72	2.036	-1.177	4
H_2C=CHOH *syn*	1.080	1.359	110.99	2.016	-1.138	5
HCOOH *cis*	1.098	1.192	123.95	2.021	-1.152	6
HCOOH *trans*	1.092	1.198	125.04	2.032	-1.174	6
**H**$COOCH_3$	1.093	1.200	124.90	2.033	-1.176	7
$HCOOCH_3$ s	1.083	1.434	105.57	2.016	-1.104	7
$HCOOCH_3$ a	1.087	1.434	110.14	2.076	-1.104	7
$(CH_3)_2O$ s	1.086	1.406	107.52	2.019	-1.084	5
$(CH_3)_2O$ a	1.095	1.406	111.14	2.071	-1.084	5
CH_3CH_2OH *trans*	1.094	1.422	110.61	2.077	-1.110	1

References

1. J. Demaison, M. Herman, J. Liévin. CCSD(T)_ae/wCVQZ + MP2/cc-pV5Z – MP2/cc-pVQZ. The equilibrium OH bond length. Rev. *Phys. Chem*. **2007**, *26, 391-420.*
2. W.J. Morgan, D.A. Mathhews, M.Ringholm, J. Agarwal, J.Z. Gong, K. Ruud, W.D. Allen, J.F. Stanton, H.F. Schaefer III. Geometric energy derivatives at the complete basis set limit: application to the equilibrium structure and molecular force field of formaldehyde. *J. Chem. Theory Comput*. **2018**, *14, 1333-1350.*
3. J. Demaison, J.E. Boggs, H.D. Rudolph. Ab initio anharmonic force field and ab initio and experimental equilibrium structures of formyl chloride. *J. Mol. Struct.* **2004**, *695-696, 145-153.*
4. L. Margulès, J. Demaison, J.E. Boggs. Equilibrium C-F bond length and the structure of formyl fluoride, difluorocarbene, monofluoromethylene, and difluoromethane. *J. Phys. Chem. A* **1999**, *103, 7632-7638.*
5. N. Vogt, J. Demaison, J. Vogt, H.D. Rudolph. Why it is sometimes difficult to determine the accurate position of a hydrogen atom by the semiexperimental method: structure of molecules containing the OH or the CH_3 group. *J. Comput. Chem.* **2014**, *35, 2333-2342.*
6. J. Demaison, M. Herman, J. Liévin. Anharmonic force field of *cis*- and *trans*-formic acid from high-level *ab initio* calculations, and analysis of resonance polyads. *J. Chem. Phys*. **2007**, *126, 164305.*
7. J. Demaison, L. Margulès, I. Kleiner, A.G. Császár. Equilibrium structure in the presence of internal rotation: A case study of cis-methyl formate. *J. Mol. Spectrosc*. **2010**, *259, 70-79.*
